# Supplementary material for: Genomic Sequence Diversity and Population Structure of Saccharomyces cerevisiae Assessed by RAD-seq
Source: G3 (Bethesda). 2013 Oct 11;3(12):2163–71. doi: 10.1534/g3.113.007492 (PMC3852379; doi:10.1534/g3.113.007492)
Supplement: Supporting Information [file supp_3_12_2163__index.html]

Genomic Sequence Diversity and Population Structure of Saccharomyces cerevisiae Assessed by RAD-seq — Supporting Information 

# Genomic Sequence Diversity and Population Structure of *Saccharomyces cerevisiae* Assessed by RAD-seq

## Supporting Information for Cromie *et al.*, 2013

**Files in this Data Supplement:**

- Supporting Information - Figures S1-S3, Files S1-S2, and Tables S1-S3 (PDF, 630 KB)
- Figure S1 - Population ancestry of strains inferred by InStruct. (PDF, 149 KB)
- Figure S2 - Linkage disequilibrium as a function of physical distance. (PDF, 139 KB)
- Figure S3 - RAD-seq neighbor-joining tree of the 38 *S. cerevisiae* strains used in both this study and a previous population analysis that used whole genome sequencing (compare to Liti *et al.* 2009 Figure 1C). (PDF, 83 KB)
- File S1 - Matrix of polymorphic sites (.txt, 3 MB)
- File S2 - Neighbor-joining tree of 262 *S. cerevisiae* strains based on multiple alignment of 116,880 bases (.zip, 4 KB)
- Table S1 - Strains used in this study, with population assignments inferred by InStruct (.xlsx, 87 KB)
- Table S2 - Populations inferred using InStruct and summary statistics (.xlsx, 44 KB)
- Table S3 - Fit of the population structure model as a function of the number of populations (.xlsx, 182 KB)
