# Supplementary material for: Genomic Sequence Diversity and Population Structure of Saccharomyces cerevisiae Assessed by RAD-seq
Source: G3 (Bethesda). 2013 Oct 11;3(12):2163–71. doi: 10.1534/g3.113.007492 (PMC3852379; doi:10.1534/g3.113.007492)
Supplement: Supporting Information [file supp_g3.113.007492_007492SI.pdf]

G3·Genes | Genomes | Genetics

Genomic Sequence Diversity and Population Structure of *Saccharomyces cerevisiae* Assessed by RAD-seq

Gareth A. Cromie [*,1], Katie E. Hyma[§,1], Catherine L. Ludlow[*], Cecilia Garmendia-Torres[*], Teresa L Gilbert[2], Patrick May[*,‡], Angela A. Huang[†], Aimée M. Dudley[*], Justin C. Fay[§§]

[*] Institute for Systems Biology, Seattle, WA, USA
[§] Bioinformatics Facility (CBSU), Institute for Biotechnology, Cornell University, Ithaca, NY, USA
[‡] Luxembourg Centre for Systems Biomedicine, University of Luxembourg, Esch-sur-Alzette, Luxembourg
[†] University of Pennsylvania, PA, USA
[§§] Department of Genetics, Washington University, St. Louis, MO, USA
[1] These authors contributed equally to this work.
[2] Deceased

Running Title:

Population Structure of *Saccharomyces cerevisiae*

Keywords:

RAD-seq, yeast, phylogenetics, population structure, genetic diversity

Corresponding Authors:

Aimée M. Dudley
Institute for Systems Biology
401 Terry Avenue North
Seattle, WA 98109
Email: aimee.dudley@gmail.com
Tel: (206) 732-1214

Justin C. Fay
Washington University School of Medicine
4444 Forest Park Pkwy
St. Louis, MO 63108
Email: jfay@genetics.wustl.edu
Tel: (314) 747-1808

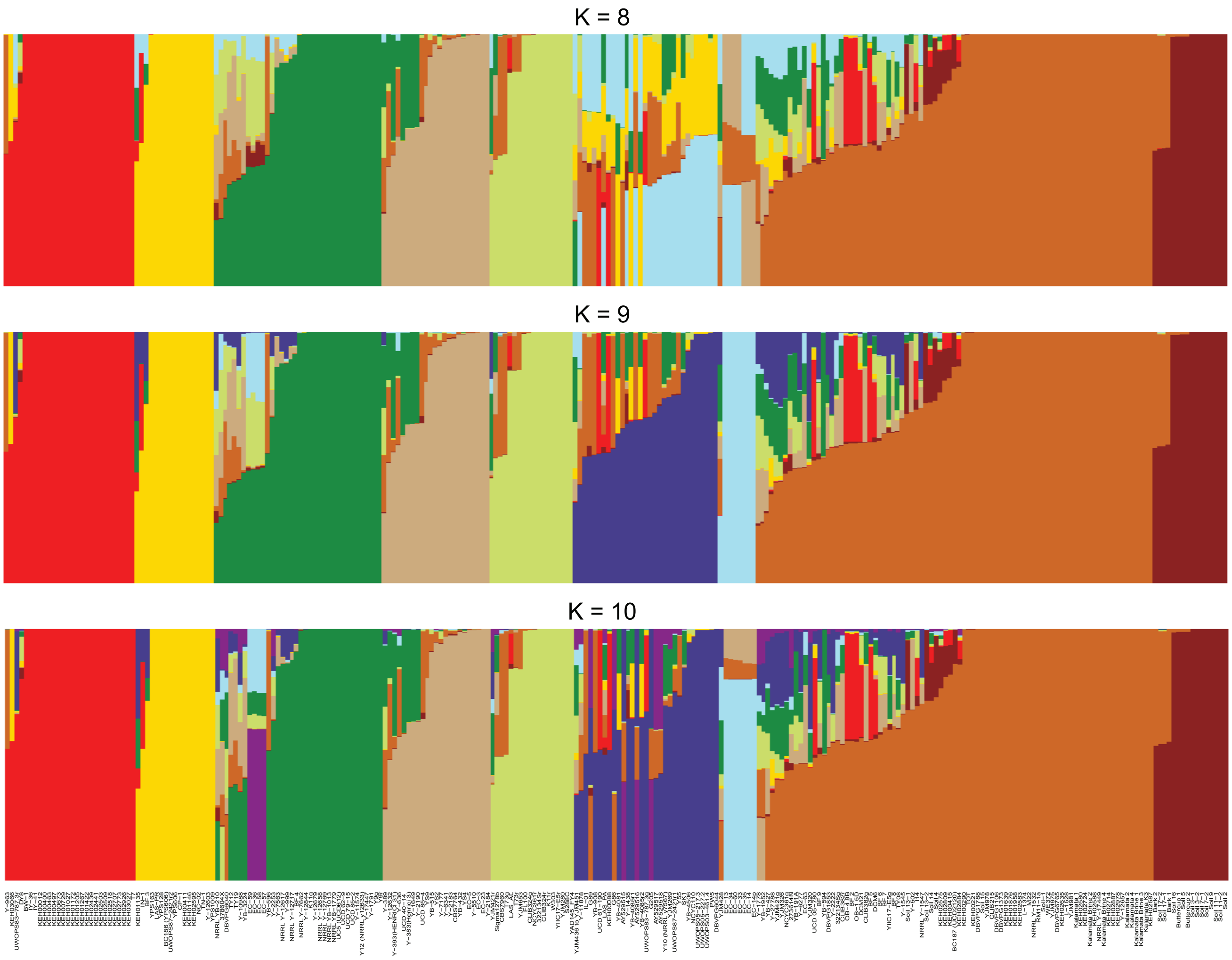

Figure S1 Population ancestry of strains inferred by InStruct. Populations are color-coded and the proportion of population ancestry assigned to each strain is indicated by bar height. Strain ancestry is shown assuming 8, 9 and 10 populations (K), with the order of strains based on K = 9 and color-coding of major populations matching that of K = 9.

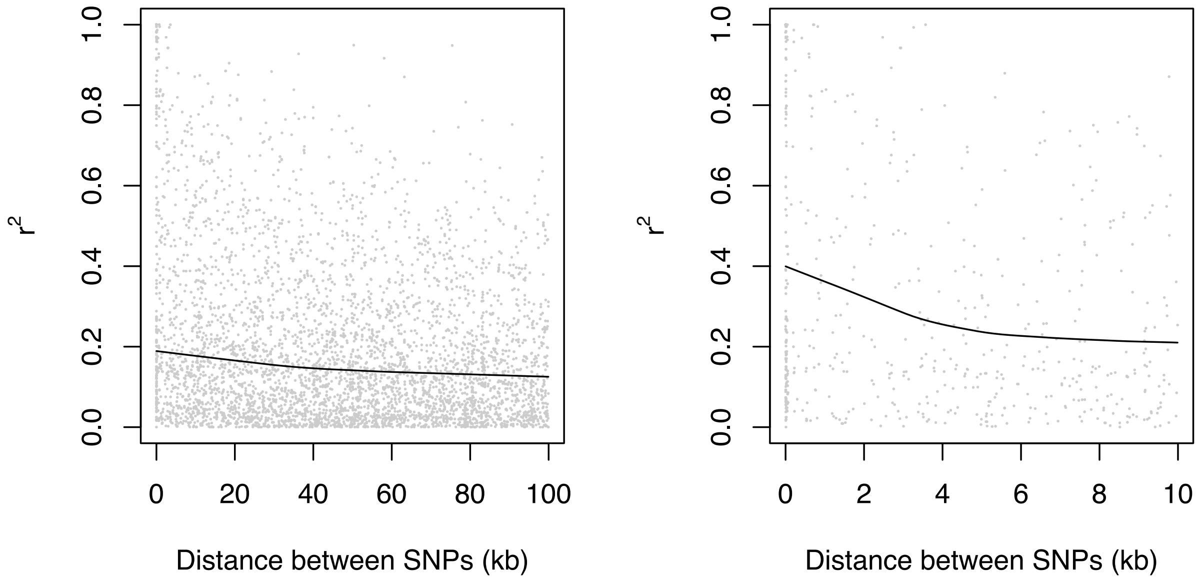

Figure S2 Linkage disequilibrium as a function of physical distance. Points show the square of the correlation coefficient (r2) between each pair of 759 common SNPs as a function of distance for sites within 100 kb of one another (A) and for sites within 10 kb of one another (B).

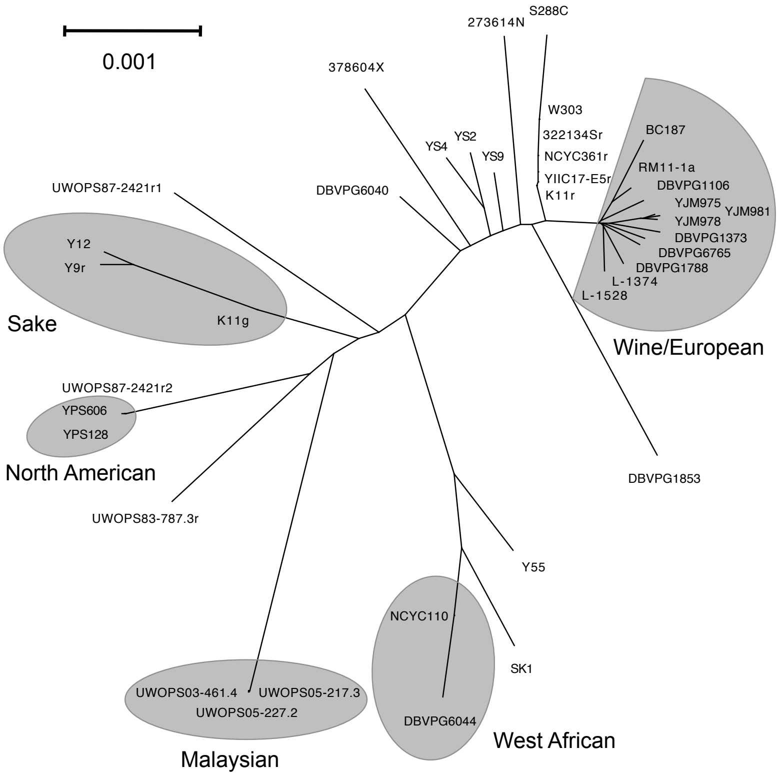

Figure S3 RAD-seq neighbor-joining tree of the 38 *S. cerevisiae* strains used in both this study and a previous population analysis that used whole genome sequencing (compare to LITI *et al.* 2009 Figure 1C). Branch lengths are proportional to sequence divergence measured as P-distance. Scale bar indicates 10 polymorphisms/ 10 kb of sequence. The 2 divergent positions for strain K11 are likely caused by mislabeling of the strain used for the "K11r" sequencing. Strains comprising the 5 lineages identified in LITI *et al.* 2009 have been labeled (North America, Sake, Malaysian, West African, Wine/European).

Supporting Data and Tables

Available for download at http://www.g3journal.org/lookup/suppl/doi:10.1534/g3.113.007492/-/DC1

File S1 Matrix of polymorphic sites. The matrix consists of 5,868 bi-allelic sites (columns) and 262 strains (rows) with column labels indicating the chromosome number and position separated by a period. Genotypes are represented by 0 or 2 for homozygotes, 1 for heterozygotes and -9 for missing data. Entries are comma delimited.

File S2 Neighbor-joining tree of 262 *S. cerevisiae* strains based on multiple alignment of 116,880 bases. This tree is a version of Figure 1 that includes strain labels and the maximum group membership from Figure 2 and is in Newick format to allow visualization with phylogenetic tree viewing software.

Table S1 Strains used in this study, with population assignments inferred by InStruct.

Table S2 Populations inferred using InStruct and summary statistics.

Table S3 Fit of the population structure model as a function of the number of populations.
