# Supplementary material for: Genomic Sequence Diversity and Population Structure of Saccharomyces cerevisiae Assessed by RAD-seq
Source: G3 (Bethesda). 2013 Oct 11;3(12):2163–71. doi: 10.1534/g3.113.007492 (PMC3852379; doi:10.1534/g3.113.007492)
Supplement: Supporting Information [file supp_g3.113.007492_FigureS2.pdf]

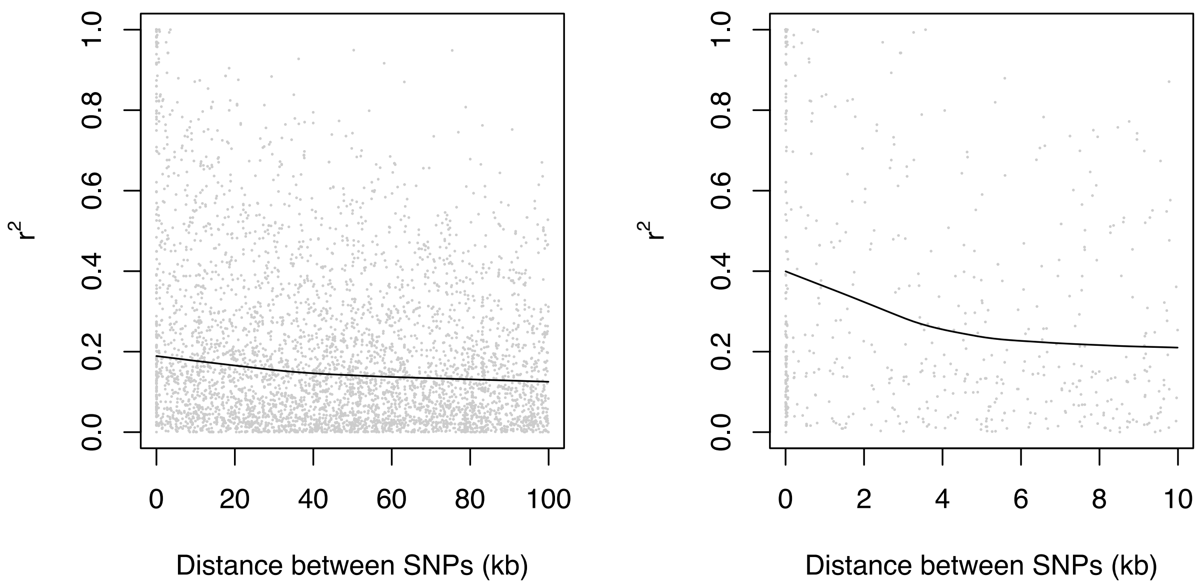

Figure S2 Linkage disequilibrium as a function of physical distance. Points show the square of the correlation coefficient (r2) between each pair of 759 common SNPs as a function of distance for sites within 100 kb of one another (A) and for sites within 10 kb of one another (B).
