# Supplementary material for: Genomic Sequence Diversity and Population Structure of Saccharomyces cerevisiae Assessed by RAD-seq
Source: G3 (Bethesda). 2013 Oct 11;3(12):2163–71. doi: 10.1534/g3.113.007492 (PMC3852379; doi:10.1534/g3.113.007492)
Supplement: Supporting Information [file supp_g3.113.007492_FigureS1.pdf]

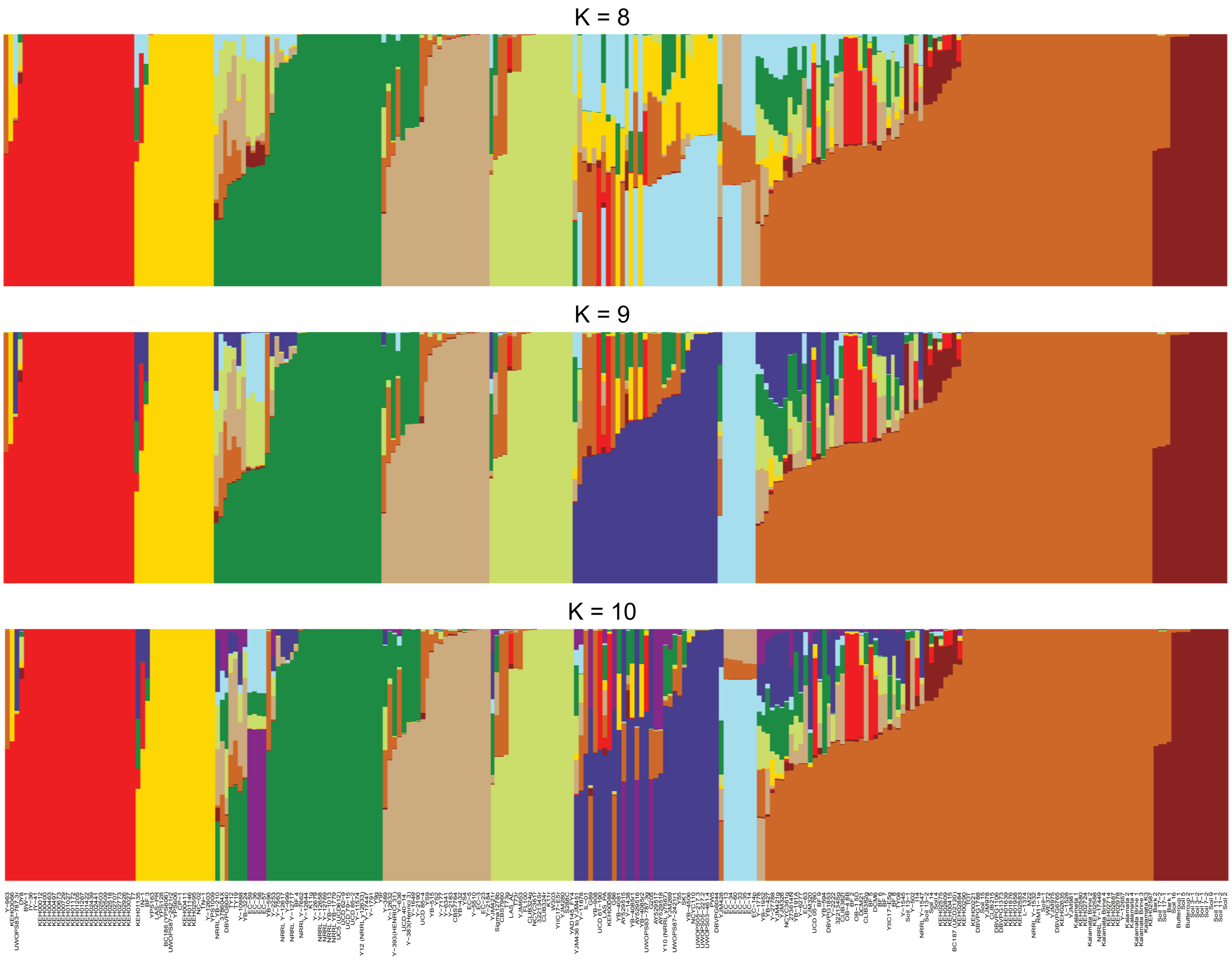

Figure S1 Population ancestry of strains inferred by InStruct. Populations are color-coded and the proportion of population ancestry assigned to each strain is indicated by bar height. Strain ancestry is shown assuming 8, 9 and 10 populations (K), with the order of strains based on K = 9 and color-coding of major populations matching that of K = 9.
