# Supplementary material for: Genomic Sequence Diversity and Population Structure of Saccharomyces cerevisiae Assessed by RAD-seq
Source: G3 (Bethesda). 2013 Oct 11;3(12):2163–71. doi: 10.1534/g3.113.007492 (PMC3852379; doi:10.1534/g3.113.007492)
Supplement: Supporting Information [file supp_g3.113.007492_FigureS3.pdf]

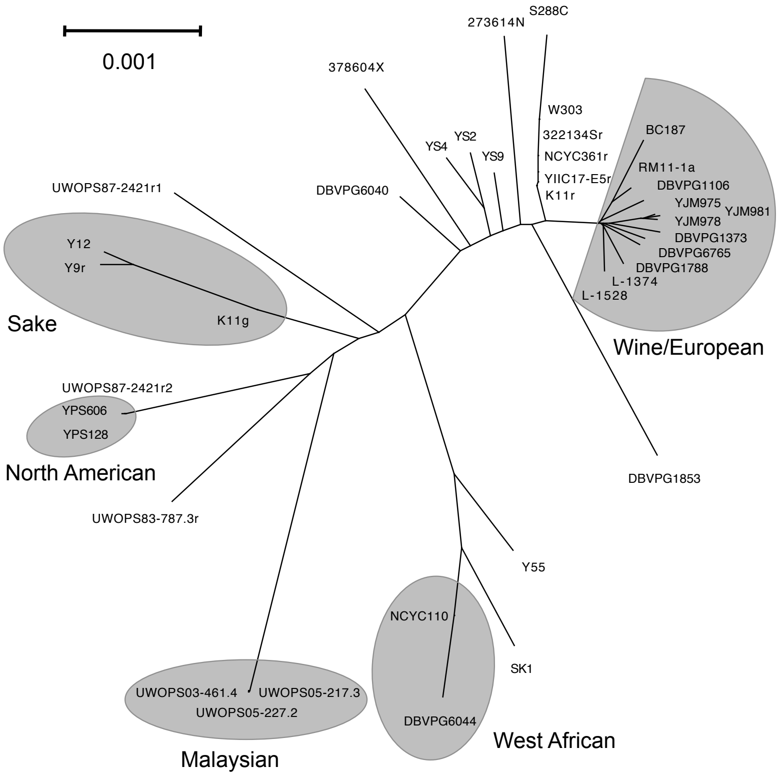

Figure S3 RAD-seq neighbor-joining tree of the 38 *S. cerevisiae* strains used in both this study and a previous population analysis that used whole genome sequencing (compare to LITI *et al.* 2009 Figure 1C). Branch lengths are proportional to sequence divergence measured as P-distance. Scale bar indicates 10 polymorphisms/ 10 kb of sequence. The 2 divergent positions for strain K11 are likely caused by mislabeling of the strain used for the "K11r" sequencing. Strains comprising the 5 lineages identified in LITI *et al.* 2009 have been labeled (North America, Sake, Malaysian, West African, Wine/European).
